# *“Oh-oh, the others are standing up... I better do the same”.* Mixed-method evaluation of the implementation process of ‘Take a Stand!’ - a cluster randomized controlled trial of a multicomponent intervention to reduce sitting time among office workers

**DOI:** 10.1186/s12889-020-09226-y

**Published:** 2020-08-08

**Authors:** Ida H. Danquah, Stine Kloster, Janne S. Tolstrup

**Affiliations:** National Institute of Public Health, University of Southern Denmark, Studiestræde 6, 1455 Copenhagen, Denmark

**Keywords:** Sedentary behaviour, Workplace, Sedentary work, Randomized controlled trial, Process evaluation

## Abstract

**Background:**

Multicomponent workplace-based interventions aimed at reducing sitting time among office workers are becoming increasingly popular. ‘Take a Stand!’ was such an intervention, reducing sitting time by 71 min after 1 month and 48 min after 3 months. However, it is unclear how the implementation process of ‘Take a Stand!’ affected these results. The present study explored how individual factors and organizational context influenced implementation and effect in ‘Take a Stand!’

**Methods:**

This was a mixed-methods study, combining data from interviews, questionnaires and accelerometers. Directed content analysis was used for analysing interviews with participants, ambassadors and managers from the 10 intervention offices in the ‘Take a Stand!’ study. Categories for analysis were taken from *Framework for Evaluating Organizational-level Interventions*. Interview data were combined with questionnaire and activity data, and multilevel analysis was undertaken to assess how changes in sitting time varied depending on the assessed factors. In addition, interview data were used to underpin results from the multilevel analysis.

**Results:**

Concurrent institutional changes were found to be a barrier for the intervention by ambassadors, while participants and managers did not find it to be an issue. Management support was consistently highlighted as very important. Participants evaluated ambassadors as being generally adequately active but also, that the role had a greater potential.

The motivational and social aspects of the intervention were considered important for the effect. This was supported by regression analyses, which showed that a strong desire to change sitting time habits, strong motivation towards the project, and a high sense of collective engagement were associated to less sitting time at 3 months of about 30 min/8 h working day compared to participants with low scores. Influence from other participants (e.g. seeing others raise their tables) and the use of humour were continuously highlighted by participants as positive for implementation. Finally, the intervention was found to influence the social climate at the workplace positively.

**Conclusion:**

Individual motivation was related to the sitting time effect of ‘Take a Stand!’, but the organizational culture was relevant both to the implementation and effect within the office community. The organizational culture included among others to ensure general participation, to uphold management and peer-support, and maintain a positive environment during the intervention period.

**Trial registration:**

ClinicalTrials.gov, NCT01996176. Prospectively registered 21 November 2013.

## Background

Sitting for long periods has been associated with adverse health outcomes; e.g. all-cause mortality and cardiovascular disease [1, 2]. The workplace is an obvious setting for intervention as many adults accumulate long periods of sitting during working hours [3, 4] and as they can be reached simultaneously [5]. Furthermore, offices constitute a specific context, which offers both advantages and challenges to targeting sitting time: e.g. employer-employee relationships and social support or peer pressure amongst colleagues [6, 7]. These circumstances should be considered when evaluating the implementation and effects of any intervention.

Nielsen et al. [8] address the need to understand how and why interventions work (or not) in order to be able to transfer interventions to practice. They therefore suggest looking at processes that influence intervention outcomes, such as participants’ attitudes and the role of managers, and link these processes to the effectiveness of the intervention: this is seldom done in organizational intervention research [8].

Nielsen & Randall [9] have proposed a model for evaluating organizational interventions that comprises of three levels of factors that influence their outcome: i.) intervention context (e.g. the organizational culture and events during the intervention phase); ii.) intervention design and implementation (e.g. the initiation of the intervention and the role of key stakeholders); and iii.) participants’ mental model (e.g. participants’ appraisal of the intervention and to what degree they share mental models). The framework has been designed to evaluate organizational-level interventions and is thus preferable to other evaluation models [9]. This model has also been used by others to evaluate a physical activity promoting intervention at the workplace [10].

A review of studies from Australia, the UK and the USA on barriers to, and facilitators of reducing workplace sitting identified elements such as work pressure, and social norms around movement, as relevant to consider when implementing interventions [11]. However, these also emphasized the need for future studies in more countries, and that included managerial perspectives [11]. Thus, to contribute to this body of knowledge, we evaluated the implementation of the Danish intervention ‘Take a Stand!’ within the above-mentioned framework for evaluation organizational interventions.

The aim of this study was to explore how factors during the implementation process influenced implementation and the effect size of the ‘Take a Stand!’ sitting time intervention towards office workers. This was done using a mixed-methods approach, and included both statistical analysis of changes in sitting time and relevant factors among the 173 participants in the intervention group, and interviews with a total of 58 participants, ambassadors and managers taking part in the project.

## Methods

### Study population and effects on sitting time

The cluster-randomized controlled trial ‘Take a Stand!’ aimed to reduce sitting time among office workers. Details on the included offices, inclusion and exclusion criteria, recruitment and randomization are reported elsewhere, together with the main results [12, 13]. In brief, the trial was conducted at four workplaces, three public and one private, in Denmark and Greenland from November 2013 to June 2014 with a total of 317 participants from 19 offices, ranging in numbers from 6 to 33 participants (mean = 17). Offices were randomized within each workplace for intervention or control at a ratio of 1:1. In this study, we included participants from the intervention group only (173 participants from 10 clusters), as interviews and questions regarding implementation were obtained only in the intervention group.

Eligible individuals were ≥ 18 years, worked > 4 days/week and were not pregnant, sick or disabled so their ability to stand or walk was affected. All participants had sit-stand desks prior to inclusion.

The main effects on accelerometer-measured sitting time was a reduction of 71 min/8 h working day after 1 month and 48 min after 3 months in the intervention group compared to the control group. Sitting was mainly replaced by standing, but the number of steps and breaks from sitting increased as well. Sitting time during leisure and time spent on physical activity did not change [12].

The study was prospectively registered at www.clinicaltrials.gov (NCT01996176) and approved by The Ethics Committees of The Capital Region of Denmark (H-6-2013-005) and Committee of Research Ethics in Greenland (project 20,914–3, id: 2014–095402). Procedures were designed in accordance with the Helsinki Declaration.

### The intervention

Details on the ‘Take a Stand!’ intervention have been published previously [12], but briefly, the main parts were: a.) appointment of ambassadors at each office and ensuring management support; b.) environmental changes, e.g. installing high meeting tables; c.) a lecture on sedentary behaviour and health; d.) a workshop aiming at ensuring local adaptation at individual, office and workplace level by individual and collective goal setting; and e.) optional weekly e-mails and biweekly text messages. Throughout the intervention, there were four broad strategies to reduce sitting: using the sit-stand desk actively; breaking up prolonged periods of sitting; having standing and walking meetings; and setting collective goals at office level. These strategies were presented together with concrete examples at the workshops, provided the framework for participants’ goal setting, and were repeated in e-mails and text messages. Control participants were instructed to behave as usual.

Fidelity to the intervention was high, based on information from observations and questionnaires. For dose delivered, all five intervention-components were implemented similarly at all four workplaces. While dose received generally showed high levels for all intervention components; 86% felt management supported the project; 79% knew where to have standing meetings; 76% participated in the workshops; and 73% signed up for the weekly e-mails [12].

### Framework for analysis

**Fig. 1:**
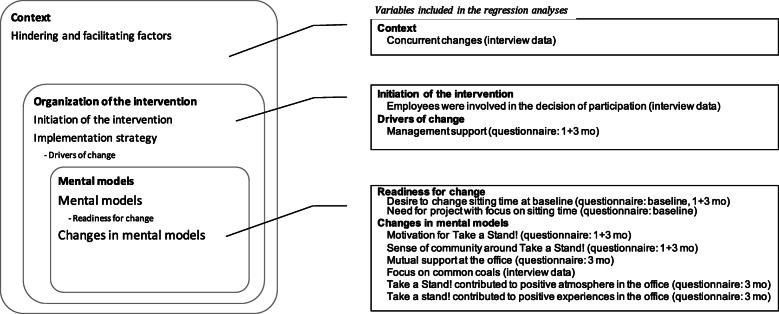
Modified version of the *Framework for Evaluating Organizational-level Interventions* by Nielsen & Randall [9]. The modified figure (to the left) shows only factors assessed in this study factors. The associated variables included in the regression analyses are displayed to the right. In brackets are time and mode of assessment; questionnaire data from baseline, 1-month or 3-month follow-up or interview data converted for statistical use

To evaluate the implementation process of the intervention and uncover how the intervention worked in different groups, a framework was selected to direct the process evaluation. The framework by Nielsen & Randall considers factors from the intervention context, factors related to the initiation and implementation of the intervention, and the mental models of the participants. Based on the framework, we have assessed a number of factors, as shown in Fig. 1 and further described below.

Context considers how different hindering and facilitating factors influence intervention outcome. Context is divided into omnibus and discrete context. Omnibus context considers the different characteristics of the participants, and has been analysed elsewhere [14]. This article will thus focus on factors in the discrete context, namely concurrent changes – events taking place at the workplace during the intervention period that affected the outcome.

Organization of the intervention includes how it is initiated and implemented. This includes participant involvement in initiating the intervention, and the role of managers and ambassadors as drivers of change.

Finally, the mental models consider the social setting and interactions within the office in relation to the intervention. This level includes factors like motivation, perceived need for the intervention, and whether participants share mental models around the intervention. More specifically, this was assessed as a sense of community and mutual support between participants in relation to the project.

### Data collection

To gain knowledge of the different factors in the model, multiple data sources are included in the article [8, 15]: 1. questionnaires to all participants (including managers and ambassadors); 2. interviews to assess the perspectives of participants, ambassadors and managers not covered by the questionnaire; and 3. accelerometers to assess the effect of the intervention.

### Questionnaire data

Questionnaire data were web-based and collected at baseline and at 1- and 3-months follow-up. Background information was recorded at baseline, while variables included in the regression analysis were recorded at different time points as follows (Fig. 1): Management support (1 and 3 months), desire to change sitting (baseline, 1 and 3 months), need for a project with focus on sitting time (baseline), motivation for ‘Take a Stand!’ (1 and 3 months), sense of collective engagement concerning ‘Take a Stand!’ (1 and 3 months), mutual support at the office (3 months), ‘Take a Stand!’ has contributed to a positive atmosphere (3 months), ‘Take a Stand!’ has contributed to positive experiences in the office (3 months).

### Interview data

Interview data was obtained from focus groups and semi-structured interviews conducted with participants, ambassadors and managers from each of the 10 intervention clusters.

Participants were interviewed in focus groups. At each office, one focus group was conducted with 2–5 participants, resulting in 11 focus groups with 33 participants in total (at one office, two smaller focus groups were set up due to practical requirements). Ambassadors helped recruit participants for focus groups. Focus groups lasted between 18 and 58 min. In addition, all ambassadors were interviewed either alone or together (if offices had appointed more than one ambassador) resulting in 11 interviews with a total of 15 participants. These interviews lasted between 11 and 70 min. Finally, all managers were interviewed, resulting in 9 interviews with 10 participants lasting between 12 and 45 mins.

All 31 interviews and focus groups took place shortly after the last follow-up measure at 3 months. Interviews and focus groups took place during working hours and at the workplace, except for 5 manager interviews, 4 interviews with ambassadors and 2 focus groups, which, due to necessity, were conducted by telephone.

The interviews followed a semi-structured interview-guide designed using questions from the framework by Nielsen & Randal [9], with a slightly different focus for participants, ambassadors and managers. Themes covered included: the workplace context (e.g. working conditions and changes during the project period); drivers of change (e.g. what motivated participants of ‘Take a Stand!’); and mental models (e.g. how they changed their work routines). Additionally, managers and ambassadors were asked about their roles in the project. The specific themes for each interview and examples of questions are displayed in Table 1.

**Table 1:** Overview of the number of participants, duration, themes and examples of question-formulations in focus groups and interviews

	Details on interviews	Themes	Example of questions
**Focus groups**	11 focus groups 2–5 participants in each 33 participants in total 18–58 min	Motivation	*Write down a concrete experience or activity in relation to ‘Take a Stand!’ – and tell the others about it.*
		Evaluation of elements	*Which elements of the intervention did you use the most? And which elements did not work?*
		Concurrent changes	*How did concurrent changes or projects affect participation in ‘Take a Stand!’*
		Resistance	*How did it affect you if your colleagues did not participate in the intervention?*
		Ethics and responsibility	*How do you feel about the workplace intervening into the health of employees?*
		Impact	*How has ‘Take a Stand!’ influenced the workplace?*
		Future	*How could you continue working on sitting time at the workplace?*
**Ambassador interviews**	11 interviews 1–3 participants in each 15 participants in total 11–70 min	Readiness for change	*How could I know that this is a workplace ready for change?* *How did you work with health previously?*
		Ethics and responsibility	*Who made the decision to participate in ‘Take a Stand!’?*
		Motivation and support *(Important)*	*What did you do to motivate your colleagues during ‘Take a Stand!’* *How did you feel being the frontrunner?*
		Evaluation of elements	*Which elements of ‘Take a Stand!’ fitted the best to your everyday work?* *And which did not?*
		Resistance	*When did you feel a less positive atmosphere in relation to ‘Take a Stand!’?*
		Concurrent changes	*How did concurrent changes or projects affect participation in ‘Take a Stand!’*
		Impact	*How has ‘Take a Stand!’ influenced the workplace?*
		Future	*How could you continue working on sitting time at the workplace? What do you think will happen from now on?*
**Manager interviews**	9 interviews 1–2 participants in each 10 participants in total 12–45 min	Readiness for change	*How could I know that this is a workplace ready for change?* *How did you work with health previously?*
		Ethics and responsibility	*According to you, to what degree is the workplace responsible for the health of the workers?* *Who made the decision to participate in ‘Take a Stand!’?*
		Motivation and support	*How did you support your employees during ‘Take a Stand!’?* *When did something unexpected happen?*
		Evaluation of elements *(Very brief)*	*Which elements of ‘Take a Stand!’ fitted the best to your everyday work?* *And which did not?*
		Resistance	*When did you feel a less positive atmosphere in relation to ‘Take a Stand!’?*
		Concurrent changes	*How did concurrent changes or projects affect participation in ‘Take a Stand!’*
		Impact	*How has ‘Take a Stand!’ influenced the workplace?*
		Future	*Will you continue to work on reducing sitting time?*

### Activity measures

Activity was recorded at baseline and after 1 and 3 months, by an ActiGraph GT3X+ accelerometer worn on the front of the thigh 24 h/day for 5 days (Monday-Friday). During this period, participants kept a log of sleeping and working hours. Data were processed using Acti4 software, which is found to have high sensitivity and specificity for the thigh-mounted ActiGraph [16–18]. Acti4 compiles total minutes spent sitting/reclining, standing, walking, climbing stairs, running and cycling. To be eligible, a day had to include > 4 h. of work or > 4 h. of leisure. Further details on the activity monitor and data processing can be found elsewhere [12].

### Data analysis

#### Qualitative analysis

All interviews were transcribed verbatim and imported into NVivo12 (QSR International Pty Ltd., 2018) for analysis. Directed content analysis was used [18] to group findings into nodes based on categories from the framework for process evaluation by Nielsen & Randall [10] as displayed in Fig. 1. Each node was then summarized into a short text describing the theme. Coding was done by the first author (IHD). For validation purposes a group of experienced qualitative researchers at the National Institute of Public Health, Denmark, read one of the interviews and gave feedback on the analysis and content. These discussions confirmed several of the findings from the main analysis.

#### Statistical analysis

Analyses were conducted using STATA/IC-14.0 (StataCorp, College Station, TX, USA). Multilevel mixed-effects linear regression was used with sitting time at 3 months follow-up as outcome, taking baseline sitting into account. Then all factors of concern (listed in Fig. 1) were tested one by one in the model. All models, which included interview data generated at cluster level, included a random intercept to account for this. Accordingly, the equation for the statistical model was:

$$ \upmu \mathrm{ij}=\alpha +\upbeta 1\times \mathrm{X}+\upbeta 2\times \mathrm{workplace}+\upbeta 3\times \mathrm{baselineworkplacesitting}+\Upsilon \mathrm{participant} $$

where μij is sitting time at 3 months for person i in workplace j and X is the factor of interest.

### Integration of methods

The different methods were integrated at several stages of the study. Using the six forms of integration defined by Frederiksen [19], points of integration are described in the following:

Theoretical integration took place as all methods sought to describe parts of the same theoretical model by Nielsen and Randall [9].

Data integration took place in the data processing phase, as part of the interview data resulted in variables, which were included in the statistical analysis. The following themes were classified into categories (yes/no):

- Concurrent institutional changes (coded ‘yes’ if participants/managers/ambassadors talked about organizational or environmental changes during the project period).
- Employees were involved in the decision of participation (coded ‘yes’ if participants described participation in the decision to join the project).
- Active ambassador (coded ‘yes’ if participants described an active ambassador, regardless of what the ambassador said).
- Focus on common goals (coded ‘yes’ if participants talked about a continuous follow-up and focus on the common goals from the workshop)
- Non-participants had negative influence on the project (coded ‘yes’ if some participants found that non-participants affected their attitude towards the project).

Finally, methods were integrated in the analysis phase as interview data were used to improve understanding of statistical findings and vice versa. Inspired by the process described by Moran-Ellis [15] and Kelle [20], the different datasets were initially analysed separately. After this, findings from statistical analysis were followed to the interview material to elaborate findings and elucidate divergence between materials.

## Results

### Population characteristics

Of the 173 participants, 105 (61%) were women, the mean age was 47 years old, and 76% had finished tertiary education (Table 2). One third rated their health as excellent or very good, 11% smoked, and 20% were classified as obese (BMI > 30). At baseline, participants had a mean sitting time of 345 min/8 h per working day and 291 min/8 h per leisure day.

**Table 2:** Participant characteristics at baseline (*n* = 173)

	N (%)	Mean (SD)^a^
***Sociodemography and health***		
Women	105 (61)	
Age, years		47 (10)
Tertiary education	130 (76)	
BMI obese (> 30)^b^	33 (20)	
Smoker	18 (11)	
Self-rated health excellent/very good	57 (33)	
***Sitting and physical activity***^***c***^		
Sitting time, min/8 h working day		345 (54)
Standing time, min/8 h working day		82 (45)
Sitting time, min/8 h leisure		291 (53)
MVPA^d^ in leisure, min/8 h leisure		45 (22)

^a^*SD* Standard Deviation

^b^*BMI* Body Mass Index

^c^Measured with Actigraph attached on thigh (*n* = 162)

^d^*MVPA* Moderate-to-Vigorous Physical Activity (total time spent walking fast (> 100 steps/min), running, climbing stairs, rowing and cycling

### Context

#### Concurrent institutional changes

In four out of ten offices, concurrent institutional changes were described during the interviews. These included organizational changes with new department structures, new offices, changes in management and a series of redundancies. However, in the multilevel analysis, we found no association between these concurrent events and their effect on sitting time: participants from offices with concurrent changes were sitting 23 min/8 h working day (CI95% − 10; 55, *p* = 0.171) more compared to participants from offices without concurrent changes (Table 3).

**Table 3:** Association between factors during implementation and sitting time at 3 months follow-up compared to baseline. Intervention group only (*n* = 173).

	Variable	When	Category	n (%)	Coef.	95% CI		p
Context	Concurrent institutional changes	Interviews	Yes	67 (50)	23	-10	55	0.171
Initiation of the intervention	Initiation (participants influenced initiation)	Interviews	Yes	87 (65)	5	−38	47	0.826
Drivers of change	Management support	1 month	High/very high	98 (77)	−1	−27	25	0.950
		3 months	High/very high	89 (67)	−16	−40	8	0.191
	Active ambassador	Interviews	Yes	58 (43)	5	−39	48	0.836
Readiness for change	Desire to change sitting	Baseline	Strong/very strong	65 (49)	0	−21	21	0.985
		1 month	Strong/very strong	79 (62)	**−35**	**−56**	**−15**	**0.001**
		3 months	Strong/very strong	72 (56)	**−31**	**−53**	**−9**	**0.005**
	Need for project with focus on sitting time	Baseline	Strong/very strong	63 (48)	−9	−31	12	0.393
Changes in mental models	Motivation for ‘Take a Stand!’	1 month	Strong/very strong	87 (68)	**−34**	**−55**	**−13**	**0.002**
		3 months	Strong/very strong	73 (57)	**−36**	**−57**	**−16**	**0.001**
	Sense of collective engagement concerning ‘Take a Stand!’	1 month	High/very high	64 (50)	3	−21	26	0.831
		3 months	High/very high	60 (47)	**−28**	**−50**	**−6**	**0.011**
	Mutual support at the office	3 months	Totally/partly agree	93 (72)	−17	−42	8	0.175
	Focus on common goals	Interviews	Yes	101 (75)	19	−14	51	0.255
	‘Take a Stand!’ has contributed to a positive atmosphere	3 months	Totally/partly agree	85 (66)	−14	−37	10	0.246
	‘Take a Stand!’ has contributed to positive experiences in the office	3 months	Totally/partly agree	81 (63)	−8	−31	15	0.476
	Non participants (influenced project)	Interviews	Yes	42 (31)	−3	−31	26	0.864

This might be explained by the fact that many ambassadors found that the concurrent changes were influential; *“We all agreed that the timing was unfortunate. I mean, it was during a period of intense work pressure and unrest in the organisation”* (Ambassador, B14), while participants and managers said they had no influence: *“Well, management backed it in both places, so it didn’t mean anything.”* (Focus Group, B14) and *“I don’t think it had any influence on project outcomes.”* (Manager, B14).

In a few departments, participants even described that ‘Take a Stand!’ had come at a good time because it gave them a common project across the new department, *“It may have had a positive impact, in the sense that it added something else that we could work on collectively to get to know each other better.”* (Manager, B4) and *“I think the project was good for us in our situation, with a whole new management and new departments, so we had some kind of a joint project.”* (Focus Group, B8). In this way the project became part of the social context at the workplace: *“The parts of “Take a Stand!” that we used were the more social aspects, so there was a bit more to it than just telling each other to stand up.”* (Ambassador, B8).

### Intervention

#### Initiation of ‘Take a Stand!’

During the interviews, participants from 6 out of 10 offices (representing 65% of all participants in the intervention group) stated that they were included in the decision about participating in ‘Take a Stand!’ In the multilevel analysis, we found no association between this influence and the effects (Table 3). Across offices, participants talked very differently about the initiation of the project. At some offices, very enthusiastic managers sold the project to the participants: *“The first time we were introduced to it was at a departmental meeting. Peter and Johanne said we were going to be part of something exciting. He was already very committed, and thought it was great that we’d been allowed to take part because so many were up for it.”* (Focus Group, A11). Additionally, some managers succeeded in appealing to everybody, making them feel they were able to participate: *“Our managers aren’t naturally sporty types... so I think people felt that they started on the same level as the rest of us, so that probably made a difference. If we’d been confronted with a super-fit gym bunny, people might have felt that they were being coerced into it. [...] It was sold to us in a way that made everybody feel they could take part, nobody thought, ‘Oh God, I can’t, I have osteoarthritis’.”* (Focus Group, C9). On the other hand, within some offices, particularly one, participants felt imposed on by the decision to participate in ‘Take a Stand!’, and felt a lack of information and ability to influence the decision: *“I kind of felt we were being forced into it, and weren’t really provided with proper information about why us and what it was for. It was just kind of, ‘Well, you have to do it’. I didn’t feel like I had any say in the matter*.” (Focus Group, B14).

In addition, participants from several offices expressed that they had wanted more information about ‘Take a Stand!’ and the content from the very beginning, which is in line with managers explaining that they sold the project, not as a sitting time project per se, but more as a health or wellbeing-at-work project: *“I said we were going to be part of a research project that would enhance well-being in the workplace, instil good habits and make us more active during the day, and that I thought it was really positive, and doing it would generate a lot of positive energy, make us more efficient, and enhance the well-being of individual members of staff*.” (Manager, A11).

#### Drivers of change

##### Management support

At 1 month, 120 participants (77%) felt a high/very high degree of management support towards the project. At 3 months, the number was 105 (67%). In the multilevel analysis, neither management support at 1 nor 3 months was associated with outcomes (Table 3). However, in the interviews, participants emphasized how important management support was in order for ‘Take a Stand!’ to be realized, e.g. *“I really think that the single most important aspect has been that management and colleagues backed the project.”* (Focus Group, C9). This was expanded on by explaining how managers could show their support for the project and show it was important and possible by setting a good example: *“So, our manager played a very active part in the project, and did a lot to make sure people followed up on the targets. At the very least, she was good at keeping us motivated and active.”* (Ambassador, C5).

Finally, managers could state their support physically by ensuring the right facilities: *“It is a sign of management’s support for the project that our manager earmarked funds and bought 6–8 standing desks. They’re there all the time and serve as visible signs of that backing.”* (Focus Group, C9).

Management support was crucial for participants to feel they were allowed to change their work routines and spend time on the project, *“It’s really important that management thinks it is OK to spend time on it. That they’re motivated and think it’s in our best interest, even if not necessarily theirs. That means something.”* (Ambassador, A11).

Management interviews showed that managers were very aware of this role, as they understood the need to be supportive, participating, and enthusiastic and to ensure focus on the project and the common goals, e.g.: *“Well, my role is to focus on the fact that we’re part of this project. Much of what we’ve done as a department has been based on ‘Take a Stand!’ All of the departmental meetings, one-on-one meetings, etc., that I’ve been to have taken place standing up as far as possible. And then there is the constant encouragement – ‘Remember to stand up!’”* (Manager, D7). Being the good example and inviting workers to stand was also expressed by another manager, especially when making it fun: *“I also used a bit of humour. Like when I stand up and shout ‘All rise!’ like in an American courtroom”* (Manager, D13).

The managers also emphasized the need for them to participate equally with the employees, and their roles in supporting both the ambassador and the employees: *“It requires management backing. We support it, and say that you can do it in your working hours, including spending time preparing for it and stuff like that. That’s needed, of course*.*”* (Manager, C9).

##### Ambassadors

During the interviews, participants, ambassadors and managers from 4 out of 10 offices (representing 58 persons (43%)) described an active ambassador, mentioning different initiatives and supportive actions the ambassador had carried out during the project period. However, having an active ambassador was not related to sitting time in the multilevel analysis (Table 3).

In several interviews, participants described how ambassadors made fun activities and small reminders, e.g., in one department, ambassadors put up notes on toilets and coffee machines suggesting using another one down the hallway (a11). Ambassadors described their role as motivators and as setting good examples, and many took the role of reminding participants of the project: *“If the boss forgets that you could stand up, one of the ambassadors always says, ‘Shouldn’t we stand up for some of the items on the agenda?’”* (Ambassador, A11).

Nevertheless, other participants called for more active ambassadors, however, some ambassadors described barriers in this regard. On one hand, there were barriers such as time constraints on themselves and participants *“You have to remember to do it [remind colleagues about the project], because we’re just so damn busy. It would be easy for it to be drowned out by everything else.”* (Ambassador, C9), and on the other hand, there was a fear of going too far and interfering with people’s privacy: *“I actually think it’s been difficult sometimes. Part of me thinks that, in my department, a lot of it has just happened of its own accord. And when it didn’t, it’s been a question of not interfering too much. It’s maybe best to leave people in peace, but also make sure there’s always room for them among the rest of us if they want to join in.”* (Ambassador, D13).

### Mental models

#### Participants’ readiness for change

##### Desire to change sitting and need for sitting time project

At baseline, 1 month and 3 months, participants were asked to what degree they wanted to change their sitting time during work hours. Answers at baseline had no association with outcome, but participants with a strong/very strong desire to change sitting time at 1 month reduced sitting by 35 min/8 h working day at 3 months compared to participants with less desire (CI95% -55.7; − 15.0, *p* = 0.001). Participants with a strong/very strong desire to change sitting time at 3 months reduced sitting by 31 min at 3 months compared to participants with less desire (CI95% -52.5; − 9.4, *p* = 0.005). The proportion of participants with a strong/very strong desire to change sitting increased slightly from 49% (65) at baseline to 62% (79) at 1-month follow-up, followed by a decrease to 56% (72) at 3-months. In addition, we assessed to what degree participants felt they needed a project on sitting time at baseline: 48% (63) thought this was necessary, but there was no association with outcome (Table 3).

Baseline measures were taken before participants were randomized to intervention or control and before they knew about ‘Take a Stand!’ During the final interviews, participants explained that the workshop and knowledge about the health consequences of sitting motivated them: *“It made a really big impression to be presented with the facts about what it actually means to sit down. It was really motivational.*” (Focus Group, A11).

Before ‘Take a Stand!’, participants had not considered their sit-stand desk as a means of varying their working day: *“I’ve always had sit-stand desks, I’ve just never used them to stand up.”* (Focus Group, A11). However, many participants were positively surprised how long they could stand without being tired and how easily they were able to change their sitting habits (Focus Group, A19). Some participants even described how it became more natural to stand than sit: *“I’ve also noticed that when we’re sitting down in meetings, I get antsy quickly and need to stand up.”* (Focus Group, C5).

#### Changes in mental models

##### Motivation and retention

In follow-up questionnaires at 1 and 3 months, intervention group participants were asked about their motivation for ‘Take a Stand!’ and this measure was strongly associated with sitting time at 3 months. Participants with strong/very strong motivation at 1-month follow-up were sitting − 34 min/8 h working day less than participants who answered they were only somewhat, little or not at all motivated for the project (CI95% -55; − 13, *p* = 0.002). For motivation at 3 months follow-up the difference was − 36 min (CI95% -57; − 16, *p* = 0.001). The absolute level of motivation fell from 68% (87) with strong/very strong motivation at 1 month to 57% (73) at 3 months follow-up. As mentioned, perceived management support fell from 77% at 1 month to 67% at 3 months (Table 3). During interviews, a general theme was decreased motivation over time, because project focus was replaced by other projects, old habits and usual work: *“We’ve also been at it a long time – three months. It’s been a bit of a challenge to keep it all going for so long, because we have to work as well. But that’s why we’re here!”* (Ambassador, C9).

Several participants explained how the workshops motivated them, but that over time this motivation had faded, and they would have liked more reminders of the health consequences of sitting during the period. However, some participants explained that something had changed in the way they worked, which they expected to continue: *“Have I learned new habits? Yes! Yes, I think I have.”* (Ambassador, C9).

#### ‘Take a Stand!’ in the office community

Participants with a high/very high sense of collective engagement concerning ‘Take a Stand!’ at 3 months follow-up had 28 min less sitting time at 3 months compared to participants with a lower sense of collective engagement (CI95% -49.5; − 6.4, *p* = 0.011). However, sense of collective engagement at 1-month follow-up was not associated with sitting time effect at 3 months. Mutual support at the office, focus on common goals, positive atmosphere concerning the project, and whether the project created positive experiences with colleagues, all measured at 3 months follow-up, showed no relation to sitting time at 3 months (Table 3).

Several additional variables, measured at 3 months follow-up regarding the community and atmosphere at the office, showed no relation to sitting time at 3 months. This was the case for mutual support at the office, focus on common goals, positive atmosphere concerning ‘Take a Stand!’ and whether ‘Take a Stand!’ created positive experiences with colleagues (Table 3).

Interviews revealed several relevant themes regarding social aspects of ‘Take a Stand!’: mutual influence between participants, the use of humour when implementing the project, and the important role of the project-related community at the workplace, which led to secondary social effects of the project. These themes are elaborated below.

##### Mutual influence between participants

During interviews, participants described how they were influenced by seeing their colleagues raising their desks, and were thus encouraged or reminded to do the same themselves, e.g. *“There’s a bit of a domino effect. Oh-oh, the others are standing up... I better do the same.”* (Focus Group, A19). In contrast, some participants worked in private offices or with non-participants and they often found it difficult to maintain their motivation or to remember the project: *“I think I’d have been reminded of it more if we’d been sitting together and I could have reminded others – if we were all sitting in a big, open-plan office, like. Or if I at least shared with somebody else, and we could have done it together – if she raised the desk, I’d do it too, I think.”* (Ambassador, C9). It seemed that seeing somebody else standing up was the best reminder of the project and the best cue to behavioural change.

##### Humour

Humour was mentioned in most focus groups and by most ambassadors as a mean to remind colleagues of the project without being strict. One participant talked about the ambassador and her role in the project: *“I could imagine somebody else adopting a different approach, but she did it in a positive and humorous way, so it was all good. It was not like she constantly told us what to do. Because that’s not the way it should be. I think.*” (Focus Group, A11).

Additionally, several ambassadors explained that they tried to have a humorous take on the activities: *“It’s been a matter of just walking around and saying, ‘Hello, stand up!’ and sending e-mails with fun links for exercises or something else to do.*” (Ambassador, B14). Other ambassadors admitted they would have liked to have provided more fun and encouraging activities.

##### The workplace community and secondary social effects

An important part of ‘Take a Stand!’ was the workplace community around the intervention. In questionnaires, 47–50% of participants answered they felt a high/very high sense of collective engagement concerning the project (as opposed to some degree/less degree/not at all) when asked after 1 and 3 months, and sense of collective engagement was related to reduced sitting time after 3 months (Table 3). During interviews, many participants emphasized the workplace community as one of the most important motivational factors: *“The sense of collective engagement around this has been the most positive thing.”* (Focus Group, D13).

The workplace community around ‘Take a Stand!’ was fostered by participating in the same workshops, wearing accelerometers at the same time and working towards common goals. In addition, some offices experienced secondary social effects, which enhanced the general sense of collective engagement and coherence: *“Standing together and formulating common goals, that at least was very, very positive, I think. You engage with each other differently. Again, I was new, and I got to know the others in a different way in the office because we all had the same goals.*” (Focus Group, C19).

In some offices, experiencing concurrent changes and thus new ways of working meant that ‘Take a Stand!’ even became more of a social thing than a health promotion project: “*When you add the big professional challenges that were so important during this period, then it doesn’t happen. Then it’s only in social contexts that there’s room for it.”* (Ambassador, B8). The social part of ‘Take a Stand!’ was also essential in offices where work was centered on helping the public, where participants expressed satisfaction about doing something for themselves, the workplace community, and for the public.

However, even though it was perceived as something essential, not everyone felt this sense of collective engagement: *“I probably didn’t get the same sense of community because I was sitting on my own. It didn’t really feel like something we were doing it together.”* (Ambassador, C9). This was reflected in questionnaire results in which about half of the participants reported collective engagement.

##### *Influence of non-participants*

In 3 offices, representing 43 (31%) of participants, non-participants were described as influencing the project negatively. However, there was no difference in the effect on sitting time between those who experienced negative influence of non-participants, and those who did not (Table 3).

All offices had some non-participants, who, from the descriptions in the interviews, could be divided in two groups: those who just did not want to participate and those with legitimate excuses, such as being on holiday when the project started, working part-time, or having problems standing or walking (e.g. due to pregnancy). Additionally, the measurements (anthropometric measures and wearing the accelerometer) discouraged some participants. The interviews clearly indicated that how non-participants influenced the project (or did not) varied greatly. Some non-participants joined the activities such as standing meetings or raising their desk: “*They also take part if we hold meetings standing up, and at the big meetings, we all stand up – it’s all for the sake of the common good.*” (Ambassador, C5). Meanwhile, there were others who remained seated, and who even expressed aversion towards the project: *“Somebody held a meeting where we had to stand up. Somebody else at it said, ‘Oh, is this that nonsense you’re part of?’ and those who weren’t part of the project remained firmly seated and offered no support.”* (Focus Group, C5).

In offices where this happened, both participants and managers agreed it affected the motivation towards the project negatively because the whole office was not participating and supporting each other: *“It really gets to you. It would’ve been much better if everybody had been involved and thought it was fun, because we’ve been rather split. And that’s really bad for motivation.”* (Ambassador, S16). Managers reported that these negative non-participants created a dilemma between trying to motivate them to participate, or simply excluding them from discussions of the project.

## Discussion

During the intervention period, the implementation and the sitting time effect of ‘Take a Stand!’ were influenced by factors related to context, organization of the intervention and the mental models of the participants. Main results of each level are summarized and discussed below.

### Context

Even though it was a theme for the ambassadors, participants did not find concurrent organizational changes an obstruction to the intervention; in some cases they even welcomed the intervention into a period of organizational changes. This finding is supported by the claim that organisations constitute an ever-changing environment [8]. Thus, organizational changes should be understood as a condition when implementing workplace interventions. Rather than seeking organizational stability, it is important to find the right match between the organizational context and the intervention [8]. In the current study, the intervention had a focus on common goal setting and mutual support, which fulfilled need for common, social activities in the new organizational structure of some offices.

### Organization of the intervention

Regarding the initiation of the intervention, some participants felt they were included in the decision to participate, while others felt imposed upon and unengaged. Several participants mentioned that they would have liked more information about the content of the project from the very beginning. Other studies have shown that a positive and participatory initiation of an intervention is an important driver of behavioural change [21, 22]. Furthermore, one could hypothesize that an initial negative impression of the intervention might be something participants carry with them throughout the project period, thus affecting their overall motivation. However, we found an increase in participants with a strong desire to change sitting and a strong need for a project on sitting time from the baseline to the 1-month follow-up. This change might be caused by the introductory workshop providing more information about the intervention and the health effects of sitting. Therefore, even though participants felt imposed upon, and lacked motivation in the beginning, the workshops could have engaged them, thereby removing the initial barrier. Finally, the limited information on the project was deliberate in order to prevent contamination of the control group, which is a condition in randomized controlled trials.

Participants consistently emphasized the role of the managers, which is in line with several other studies of other occupational health interventions [8, 23] and sitting time interventions specifically [21, 24, 25]. In their review, Hadgraft et al. [11] found support from managers in the form of approval of intervention activities and leading by example to be key facilitators in reducing workplace sitting, and this has been further supported by more recent studies [26].

In addition to management support, previous studies have found workplace champions play an important role during the implementation of workplace interventions [21, 23, 26–29]. However, in the present study, the role of ambassadors showed no relation to the effect of sitting, and interviews also listed mixed results. One reason for this could be that the activity level of ambassadors varied between offices, and the expectations towards ambassadors varied between participants, with some participants calling for more active ambassadors. Research on the role of change agents in workplace health promotion has highlighted the need to clarify mutual expectations between ambassadors and participants regarding actual activities and levels of ‘pushiness’ [23].

### Mental models

In general, the social element of the project played an important role - that is consistently emphasised by the literature; a supportive environment is crucial for workplace health interventions to be implemented successfully [21, 30] . More specifically, we found that a desire to change sitting time, motivation towards ‘Take a Stand!’ and sense of collective engagement concerning the project were associated with lower sitting time after 3 months. Additionally, mutual influence between participants (e.g. seeing others raising their desk) and the use of humour were constantly highlighted. The social climate at workplaces was also important during implementation of the intervention and was able to be improved through intervention activities.

Our findings on motivation are supported by other studies that highlight that a positive attitude from participants is essential for implementation [8]. During the intervention period, participants were prompted to change their behaviour and e.g. raise their desks by seeing others doing so, and this is supported by findings from other sitting time interventions [27, 30–32] and findings that workers in individual offices had higher sitting times compared to workers in shared offices [33].

In our study, participants and ambassadors pointed to the use of humour as an effective means during the intervention period, which is supported by other studies. However, although we did not find this in our study, it has also been suggested that such strategies are effective only during the initial stage [26].

Several studies have emphasized that social support from other participants is essential to intervention implementation and effectiveness [26, 30, 32, 34]. As in our study, participants in other studies have highlighted sitting interventions as a positive reason to come together at the office [26, 35]. In general, social norms about sitting when working can act as a barrier for behavioural change [21, 24], and this has also been supported by participants in ‘Take a Stand!’, who have commented that it has to be acceptable and permissible to spend time on the project. This suggests that social norms should be addressed when developing and initiating interventions in order to ensure social support.

In some offices, not all employees participated in the intervention, and some even expressed their negativity towards the project e.g. during standing meetings. In this case, non-participants may negatively influence the results of the intervention – something that has been brought up by others who suggest the need for sitting time interventions to be implemented throughout the entire workplace [26, 27, 31, 32] and be embedded in the organization and productive work [21, 36].

Looking at the results across the three levels (context, organization of the intervention and mental models), we have identified a number of promoters and barriers that are potentially relevant for future implementation. The principle promoters identified were management support, both when initiating the project and over the course of the intervention period; the social aspects of the interventions including mutual support between participants; and the use of humour and collective engagement to create a community around the intervention. As discussed above, several promoters were identified in other studies also (e.g. [11, 34]). We identified several barriers towards intervention implementation and effect across the three levels: concurrent changes at the office; being coerced to participate; lack of information about the intervention at initiation; lack of support and reminders from ambassadors, managers and co-workers; lack of motivation (both sustained and retained); and negative influence from non-participants. Several of these barriers have also been mentioned by others (e.g. a review of 32 studies identified that a significant barrier is social norms and normative beliefs about sitting [11]), confirming the importance of support from all levels.

Together, the findings of the present study are in line with findings from other evaluations of sitting time interventions, and highlight the importance of an organizational perspective (e.g. [11]): This implies that sitting time interventions should address the organizational context from intervention development, through to the implementation process in order to ensure the best possible effect on sitting time. This could be obtained by: a proper fit between intervention and the present conditions of the workplace; adequate matching of expectations towards ambassadors and between participants; addressing social norms about sitting and intervention activities; and sustained levels of management and social support.

Together with findings from other process evaluations of similar interventions, findings from the present study contribute to the body of knowledge on how these interventions work, on which factors need to be enhanced, and on potential problems to be addressed when further disseminating these interventions into real-world settings [37].

## Strengths and limitations

A major strength of the present study was the use of mixed methods, which made it possible to understand the success of interventions and to uncover associations between processes and effects [8]. Combining questionnaire and accelerometer data provided knowledge on the association between factors during the implementation and the effect on sitting time, and interviews provided insight into how these associations might work. Interviews also provided knowledge on aspects hard to measure using questionnaires, e.g. how ‘Take a Stand!’ was affected by and influenced the social climate at the office. In some cases, different data sources yielded contradictory results. For example, while we found no association between management support and the sitting time effect, interviews emphasized management support as crucial to the project. However, the reported level of management support was generally high, and this might explain why we could not find any statistical association. Another explanation may be that the questionnaire-measured level of management support is something different to that described during interviews, and thus cannot be directly compared – therefore suggesting that they should be seen as a supplement to each other.

Another strength was that participants, ambassadors and managers were interviewed, which contributed important perspectives on the results. Finally, the outcome measure, sitting time, was measured with accelerometers on the thigh, which is currently considered the most accurate method for measuring sitting time [38].

A limitation of the present study was the fact the interviews were coded by a single researcher (IHD). In order to accommodate this limitation, results were verified in other ways: results were discussed thoroughly in the author-group; a group of experienced qualitative researchers gave feedback on the analysis of one of the interviews; and findings were often confirmed in several other ways such as in data from participants, ambassadors and managers or through the mixed-methods design. However, this approach might potentially have caused some bias in the results due to the tendency in mixed-methods research to follow the more interesting findings in the material [39]. The results should therefore be interpreted with caution, and in cases where they do not confirm findings from other studies, higher quality studies should be conducted.

Even though we had a high number of participants in the trial, analysis for the present paper was restricted to the intervention group, which reduced the number of clusters and the number participants, making it harder to detect small statistical differences.

In addition to the factors assessed, others have found a relationship between social-cognitive factors and intervention effect on sitting time [40]. Thus, including factors such as perceived behavioural control and social norms could have contributed to knowledge of the prevalent processes during implementation, and of how and why the social climate at the workplace and the intervention affected each other.

## Conclusion

Management support was pointed out as very important, but we received mixed views on the consequences of concurrent changes during the intervention period and the role of the ambassadors. Desire to change sitting and motivation towards the project influenced the sitting time effect of ‘Take a Stand!’

Finally, the social element of the project was important, as a high sense of collective engagement was related to decreased sitting time, and participants highlighted mutual support, use of humour and the social community in the office as important for the project to succeed.

Altogether, when implementing sitting time interventions within the office community, the motivation of individual employees seems important, as does the organizational culture surrounding the project, ensuring general participation, management and peer support and fostering a positive atmosphere at the office during the intervention period.

## Data Availability

The dataset used during the current study is available from the corresponding author on reasonable request.

## Abbreviations

BMI: Body Mass Index
CI95%: 95% Confidence Interval
MVPA: Moderate-to-Vigorous Physical Activity
SD: Standard Deviation

## References

[1] Ekelund U, Steene-Johannessen J, Brown WJ, Fagerland MW, Owen N, Powell KE, Bauman A, Lee IM (2016). Does physical activity attenuate, or even eliminate, the detrimental association of sitting time with mortality? A harmonised meta-analysis of data from more than 1 million men and women. Lancet..

[2] Pandey A, Salahuddin U, Garg S, Ayers C, Kulinski J, Anand V, Mayo H, Kumbhani DJ, de Lemos J, Berry JD (2016). Continuous dose-response association between sedentary time and risk for cardiovascular disease: a meta-analysis. JAMA Cardiol..

[3] Thorp AA, Healy GN, Winkler E, Clark BK, Gardiner PA, Owen N, Dunstan DW (2012). Prolonged sedentary time and physical activity in workplace and non-work contexts: a cross-sectional study of office, customer service and call Centre employees. Int J Behav Nutr Phys Act..

[4] Tudor-Locke C, Leonardi C, Johnson WD, Katzmarzyk PT (2011). Time spent in physical activity and sedentary behaviors on the working day: the American time use survey. J Occup Environ Med..

[5] World Health Organization. Workplace health promotion. The workplace: a priority setting for health promotion. https://www.who.int/occupational_health/topics/workplace/. Accessed 19 July 2020.

[6] Wierenga D, Engbers LH, Van Empelen P, De Moes KJ, Wittink H, Grundemann R, van Mechelen W (2014). The implementation of multiple lifestyle interventions in two organizations: a process evaluation. J Occup Environ Med..

[7] World Health Organization / World Economic Forum: Preventing noncommunicable diseases in the workplace through diet and physical activity: WHO/World Economic Forum report of a joint event. 2008.

[8] Nielsen K, Taris TW, Cox T (2010). The future of organizational interventions: addressing the challenges of today's organizations. Work Stress..

[9] Nielsen K, Randall R (2013). Opening the black box: presenting a model for evaluating organizational-level interventions. Eur J Work Organ Psy..

[10] Wahlstrom V, Fjellman-Wiklund A, Harder M, Jarvholm LS, Eskilsson T (2019). Implementing a Physical Activity Promoting Program in a Flex-Office: IA Process Evaluation with a Mixed Methods Design. Int J Environ Res Public Health..

[11] Hadgraft NT, Brakenridge CL, Dunstan DW, Owen N, Healy GN, Lawler SP (2018). Perceptions of the acceptability and feasibility of reducing occupational sitting: review and thematic synthesis. Int J Behav Nutr Phys Act..

[12] Danquah IH, Kloster S, Holtermann A, Aadahl M, Bauman A, Ersboll AK, Tolstrup JS (2017). Take a stand!-a multi-component intervention aimed at reducing sitting time among office workers-a cluster randomized trial. Int J Epidemiol..

[13] Danquah IH, Kloster S, Holtermann A, Aadahl M, Tolstrup JS (2017). Effects on musculoskeletal pain from “take a stand!” - a cluster-randomized controlled trial reducing sitting time among office workers. Scand J Work Environ Health..

[14] Danquah IH, Tolstrup JS (2019). Does it work for everyone? The effect of the Take a Stand! Sitting-Intervention In Subgroups Defined By Socio-Demographic, Health-Related, Work-Related And Psychosocial Factors. J Occup Environ Med..

[15] Moran-Ellis J, Alexander VD, Cronin A, Dickinson M, Fielding J, Sleney J, Thomas H (2006). Triangulation and integration: processes, claims and implications. Qual Res.

[16] Skotte J, Korshoj M, Kristiansen J, Hanisch C, Holtermann A (2014). Detection of physical activity types using triaxial accelerometers. J Phys Act Health..

[17] Stemland I, Ingebrigtsen J, Christiansen CS, Jensen BR, Hanisch C, Skotte J, Holtermann A (2015). Validity of the Acti4 method for detection of physical activity types in free-living settings: comparison with video analysis. Ergonomics..

[18] Hsieh HF, Shannon SE (2005). Three approaches to qualitative content analysis. Qual Health Res..

[19] Morten F. Integration i ‘mixed methods’ forskning: Metode eller design? Metode &amp; Forskningsdesign. 2013;1(1).

[20] Kelle U. Sociological explanations between micro and macro and the integration of qualitative and quantitative. Methods. 2001;2(1) 10.17169/fqs-2.1.966.

[21] Mackenzie K, Such E, Norman P, Goyder E (2018). The development, implementation and evaluation of interventions to reduce workplace sitting: a qualitative systematic review and evidence-based operational framework. BMC Public Health..

[22] Feltner C, Peterson K, Palmieri Weber R, Cluff L, Coker-Schwimmer E, Viswanathan M, Lohr KN (2016). The effectiveness of Total worker health interventions: a systematic review for a National Institutes of Health pathways to prevention workshop. Ann Intern Med..

[23] Eskerod P, Justesen Just B, Sjøgaard G (2017). Enriching project organizations with formal change agents: health promotion projects at the workplace. Int J Manag Proj Bus..

[24] Mackenzie K, Such E, Norman P, Goyder E (2019). Sitting less at work: a qualitative study of barriers and enablers in organisations of different size and sector. BMC Public Health..

[25] Brakenridge CL, Chong YY, Winkler EAH, Hadgraft NT, Fjeldsoe BS, Johnston V, Straker LM, Healy GN, Clark BK (2018). Evaluating Short-Term Musculoskeletal Pain Changes in Desk-Based Workers Receiving a Workplace Sitting-Reduction Intervention. Int J Environ Res Public Health..

[26] Goode AD, Hadgraft NT, Neuhaus M, Healy GN (2018). Perceptions of an online 'train-the-champion' approach to increase workplace movement. Health Promot Int..

[27] Brakenridge CL, Healy GN, Hadgraft NT, Young DC, Fjeldsoe BS (2018). Australian employee perceptions of an organizational-level intervention to reduce sitting. Health Promot Int..

[28] Hopkins JM, Glenn BA, Cole BL, McCarthy W, Yancey A (2012). Implementing organizational physical activity and healthy eating strategies on paid time: process evaluation of the UCLA WORKING pilot study. Health Educ Res..

[29] Robinson M, Tilford S, Branney P, Kinsella K (2014). Championing mental health at work: emerging practice from innovative projects in the UK. Health Promot Int..

[30] Chau JY, Daley M, Srinivasan A, Dunn S, Bauman AE, Ploeg HP (2014). Desk-based workers’ perspectives on using sit-stand workstations: a qualitative analysis of the stand@work study. BMC Public Health..

[31] Graves LEF, Murphy RC, Shepherd SO, Cabot J, Hopkins ND (2015). Evaluation of sit-stand workstations in an office setting: a randomised controlled trial. BMC Public Health..

[32] Hadgraft NT, Willenberg L, LaMontagne AD, Malkoski K, Dunstan DW, Healy GN, Moodie M, Eakin EG, Owen N, Lawler SP (2017). Reducing occupational sitting: workers’ perspectives on participation in a multi-component intervention. Int J Behav Nutrition Physical Activity..

[33] Mullane SL, Toledo MJL, Rydell SA, Feltes LH, Vuong B, Crespo NC, Pereira MA, Buman MP (2017). Social ecological correlates of workplace sedentary behavior. Int J Behav Nutr Phys Act..

[34] Brakenridge CL, Healy GN, Winkler EAH, Fjeldsoe BS (2018). What do Workers do to reduce their sitting time? The relationships of strategy use and workplace support with desk-based Workers' behavior changes in a workplace-delivered sitting-reduction and activity-promoting intervention. J Occup Environ Med..

[35] Cooley D, Pedersen S, Mainsbridge C (2014). Assessment of the impact of a workplace intervention to reduce prolonged occupational sitting time. Qual Health Res..

[36] Commissaris DA, Huysmans MA, Mathiassen SE, Srinivasan D, Koppes L, Hendriksen IJ (2016). Interventions to reduce sedentary behavior and increase physical activity during productive work: a systematic review. Scand J Work Environ Health..

[37] Owen N, Healy GN, Dempsey PC, Salmon J, Timpero A, Clark BK, Goode AD, Koorts H, Ridgers ND, Hadgraft NT (2020). Sedentary behavior and public health: integrating the evidence and identifying potential solutions. Annu Rev Public Health..

[38] Ingebrigtsen JS, Stemland I, Christiansen C, Skotte J, Hanisch C, Krustrup P, Holtermann A. Validation of a Commercial and Custom Made Accelerometer-Based Software for Step Cound and Frequency during Walking and Running. Ergonomics. 2013;3(2) 10.4172/2165-7556.1000119.

[39] Bryman A (2007). Barriers to integrating quantitative and qualitative research. J Mixed Methods Res..

[40] Hadgraft NT, Winkler EA, Healy GN, Lynch BM, Neuhaus M, Eakin EG, Dunstan DW, Owen N, Fjeldsoe BS (2017). Intervening to reduce workplace sitting: mediating role of social-cognitive constructs during a cluster randomised controlled trial. Int J Behav Nutr Phys Act..

